# Perspectives of Nursing Home Residents on Restrictive Measures and Access to Medical Services During the COVID-19 Pandemic: A Qualitative Study

**DOI:** 10.3390/healthcare14131982

**Published:** 2026-07-03

**Authors:** Almudena Crespo-Martín, Domingo Palacios-Ceña, Javier Güeita-Rodríguez, Cristina García-Bravo, Elisabet Huertas-Hoyas, Jorge Pérez-Corrales

**Affiliations:** 1 Escuela Internacional de Doctorado, Research Line in Physical Therapy, Occupational Therapy, Rehabilitation and Physical Medicine, Universidad Rey Juan Carlos, Avenida Atenas, s/n, 28922 Alcorcón, Spain; a.crespo.2019@alumnos.urjc.es; 2 Department of Occupational Therapy, El Conquistador Nursing Home, 10200 Trujillo, Spain; 3 Research Group of Humanities and Qualitative Research in Health Science of Universidad Rey Juan Carlos (Hum & QRinHS), Department of Physical Therapy, Occupational Therapy, Rehabilitation and Physical Medicine, Universidad Rey Juan Carlos, 28922 Alcorcón, Spain; domingo.palacios@urjc.es (D.P.-C.); cristina.bravo@urjc.es (C.G.-B.); jorge.perez@urjc.es (J.P.-C.); 4 Research Group of Participation, Roles, Occupations and Activities for Community Transformation (PROACT), Department of Physical Therapy, Occupational Therapy, Rehabilitation and Physical Medicine, Universidad Rey Juan Carlos, 28922 Alcorcón, Spain; elisabet.huertas@urjc.es

**Keywords:** COVID-19, nursing homes, qualitative research, health services accessibility

## Abstract

**Background/Objectives**: Nursing home residents were among the most vulnerable populations during the COVID-19 pandemic, facing strict restrictive measures, limited access to medical services, and significant psychological consequences derived from institutional confinement. Despite the magnitude of these impacts, the perspective of residents themselves remain underrepresented in the qualitative literature, particularly in the Spanish context. The aim of this study was to analyze and describe the perspectives of residents in a nursing home regarding the restrictive measures adopted by the facility and their access to medical services during the COVID-19. **Methods**: An exploratory qualitative study was conducted with 24 residents of a nursing home in Cáceres, Spain. Data were collected through in-depth interviews and field notes, and analyze using inductive thematic analysis following Braun and Clarke’s framework. **Results**: Two main themes were identified: Necessary to feel safe, but unpleasant: accepting the restrictive measures (Accepting the measures; and Better safe, even if unpleasant) and Barriers to healthcare: abandonment, fear, and age-based exclusion (Neglect and abandonment by healthcare system; The residence as a “bubble” and fear of hospital transfer; and Not treated because of our age). **Conclusions**: The findings highlight the complexity of the experiences of older adults in residential care during the COVID-19 pandemic and underscore the urgent need to balance health protection with psychological well-being, dignity, and the rights of older people in future emergency responses.

## 1. Introduction

The COVID-19 pandemic had a negative impact on the health of older adults living in nursing homes, who faced mandatory confinement, restricted family visits [1,2], and severely limited access to medical care at a time of vulnerability [3]. The virus, caused by the SARS-CoV-2 virus, was first identified in Wuhan, China, in December 2019 [4], and was declared a pandemic by the World Health Organization (WHO) on 11 March 2020 [5]. This virus causes respiratory infections ranging from mild colds to severe acute respiratory distress syndrome [6]. According to the WHO, over 779 million cases of COVID-19 had been reported globally by January 2026, with Spain accounting for approximately 14 million cases [7]. Transmitted primarily through respiratory droplets and aerosols, the disease has an incubation period of 2 to 14 days, which complicated containment efforts [8]. Older adults with comorbidities, as well as individuals with chronic conditions such as diabetes, hypertension, and cardiovascular diseases, were identified as high-risk groups [9]. During the first wave of COVID-19 in Spain, 50% of all deaths occurred in nursing homes [10], largely due to risk factors such as comorbidities, advanced age, and shared living spaces, making this population particularly vulnerable [11]. As a result, nursing homes were compelled to implement measures such as restricting visits and modifying routines, which had negative physical and psychological impacts on most residents [1,2]. Additionally, during the COVID-19 lockdown, access to medical services was severely limited due to healthcare system overload [3]. Countries such as Greece, Italy, and Spain exceeded their maximum intensive care unit (ICU) bed capacity [12], and older adults were often discriminated against in their access to healthcare services on the basis of age [13].

Qualitative research has shown that social isolation and loneliness were among the most significant psychological consequences of institutional confinement in nursing homes during the pandemic [2,14,15]. Studies conducted in various national contexts have demonstrated that restrictions on family visits, limitations on activities, and the perception of being cut off from the outside world led to feelings of loneliness, sadness and fear among older adults and nursing homes residents [16,17]. However, these experiences unfolded within a broader context of structural ageism [18]. Ageism is a social determinant of health [19] that the pandemic made more visible in policy decisions and clinical practice [18]. In several cases during the COVID-19 pandemic, including in Spain, age was considered in triage decisions and resource allocation protocols, such as ICU admissions and hospital referrals from nursing homes, raising significant ethical concerns regarding age-based discrimination [20]. These practices directly conflict with rights-based frameworks, which establish that nursing home residents retain fundamental rights to dignity, autonomy, and equitable access to care, regardless of context [21].

Although the impact of COVID-19 on long-term care facilities has been extensively studied, there remains a clear gap in the literature regarding how nursing home residents experienced restrictive measures and access to healthcare services during the COVID-19 pandemic, particularly within the Spanish context. Although previous qualitative studies [2,14,15,16,17] have documented psychological consequences such as loneliness, sadness, fear, and social isolation among nursing home residents during the pandemic, fewer studies have specifically explored how residents perceived restrictive measures and limitations in access to healthcare services as interconnected experiences. Furthermore, evidence from Spain remains scarce despite the particularly severe impact of COVID-19 on nursing homes during the first wave of the pandemic. Qualitative methods are particularly suitable for exploring residents’ subjective experiences, as they provide access to perspectives that cannot be fully captured through objective measures, and can contribute to improving the quality of care [22]. Therefore, this study contributes insights from residents’ own perspectives on the strengths and weaknesses of emergency management in residential care settings, generating knowledge that can inform the development of more equitable protocols, the psychological support of residents, and the preparedness of long-term care facilities for future health crises.

The aim of this study was to analyze and describe the perspectives of residents in a nursing home regarding the restrictive measures implemented by the facility and their access to medical services during the COVID-19 lockdown.

## 2. Materials and Methods

This qualitative study was conducted in accordance with the Consolidated Criteria for Reporting Qualitative Research (COREQ) [23] and the Standards for Reporting Qualitative Research (SRQR) [24].

### 2.1. Study Design

An exploratory qualitative design based on an interpretivist paradigm was employed [25]. This type of design aims to achieve a deep and detailed understanding of a specific phenomenon through the experiences, perceptions, and behaviors of individuals who have lived it. It is primarily used when information on the phenomenon is scarce and the goal is not to test hypotheses but to generate new insights and understandings that emerge from data analysis [25].

Given the identified gap in the literature, specifically the limited evidence on how nursing home residents subjectively experienced restrictive measures and reduced access to healthcare services as interconnected dimensions of the pandemic, particularly in the Spanish context, an exploratory qualitative design was considered the most appropriate methodological choice. An exploratory qualitative approach is especially suitable for investigating phenomena that have received little attention from an inductive perspective. This type of design allows researchers to address underdeveloped areas in the literature through broad research questions, without predefined hypotheses, and by using a flexible methodological strategy that can be adapted as new insights emerge [26].

Exploratory qualitative studies typically rely on open ended data collection techniques, such as semi-structured interviews, and purposive or convenience sampling. They are grounded in inductive and iterative analytical processes aimed at identifying emerging patterns and themes. Their primary objective is to generate an initial and contextually grounded understanding of the phenomenon, providing a foundation for subsequent and more structured research [26]. This type of design has been widely used in health research, particularly during the COVID-19 pandemic, to explore the experiences of nursing home residents in complex contexts of care and restriction [27].

### 2.2. Participants and Sampling Strategy

Participants were recruited from a nursing home in Cáceres, Spain, using purposive sampling [28], selecting those who could provide relevant information. This nursing home has a total capacity of 90 places, of which 70 are privately funded and 20 are publicly funded through the Extremadura Service for the Promotion of Independence and Care for People with Care Needs. The staff includes 20 nursing assistants, 4 nurses, 1 rehabilitation specialist, 1 occupational therapist, 1 social worker, 1 pharmacy technician, 3 cooks and 6 cleaning staff. The center also works in coordination with a total of 5 physicians available at the city’s medical center.

The nursing home was selected because it provided access to residents who had experienced the full period of COVID-19 restrictions and because the research team had established access to the setting. Although this was a single-site study, the facility shares several characteristics commonly found in medium-sized Spanish nursing homes, including a mixed public–private funding model, multidisciplinary staffing, and coordination with local primary healthcare services. Therefore, while the findings cannot be statistically generalized, the characteristics of the setting may facilitate the transferability of the results to similar residential care contexts.

Inclusion criteria were: being over 65 years old, institutionalized in the nursing home during the lockdown period (February–November 2020), having preserved cognitive function (Mini-Mental State Examination score ≥ 27) [29], and having freely signed the written informed consent. This threshold was deliberately selected to ensure that participants had no evidence of even mild cognitive impairment, thereby maximizing their ability to recall, understand, and describe their experiences in depth during the qualitative interviews.

Participant recruitment was conducted by the primary researcher (A.C.-M.). A total of 30 eligible residents were approached to participate in the study. Of these, 24 agreed to participate, while 6 either declined the invitation or did not respond to the initial approach. Reasons for non-participation included lack of interest, the emotional burden associated with recalling experiences from the COVID-19 pandemic, and concerns regarding the confidentiality and dissemination of their testimonies.

### 2.3. Data Collection

Data were collected in two phases between May 2020 and December 2021. Initially, eight residents participated in unstructured interviews. The data from these interviews informed the development of a semi-structured interview guide. In the second phase, semi-structured interviews were conducted with 16 participants.

As data collection spanned different stages of the COVID-19 pandemic, including periods before and after the implementation of vaccination programmes and changes in restrictive measures, particular attention was paid during data analysis to potential temporal differences in participants’ accounts. No notable differences were identified in the main themes according to the timing of the interviews.

All data collection was complemented with researchers’ field notes [30], which served to contextualise the interpretation of emerging themes and to support researcher reflexivity throughout the data collection process, documenting the primary researcher’s reflections on her role as interviewer and potential sources of bias. These notes were regularly discussed within the research team and were reviewed alongside the interview transcripts during data analysis to provide contextual information, clarify participants’ narratives when necessary, and support the interpretation of emerging codes and themes.

The average duration of the interviews was 45 min. The semi-structured interview guide is presented in Table 1.

The number of interviews conducted aligns with the review by Turner-Bowker [31] on sample size estimation based on the principle of data saturation in qualitative studies, which indicates that saturation typically occurs between interviews 15 and 25, with 97.2% to 99.3% of relevant information emerging between interviews 20 and 25. Saturation was reached when no new meaning codes emerged across two consecutive interviews, when additional data no longer contributed greater richness or depth to the themes already identified and the information obtained had become redundant [32,33]. To confirm that saturation had indeed been achieved, four further interviews were conducted, following Moser and Korstjens’ recommendation [30] to continue interviewing for some additional interviews once the researcher considers saturation to have occurred. None of these interviews generated new codes, meaningfully extended the existing thematic structure, or added new informational content. All interviews were conducted in person and later transcribed verbatim for analysis [34].

### 2.4. Data Analysis

An inductive thematic analysis was conducted [35], which allowed for the identification, analysis, and reporting of patterns or themes within the data. The approach proposed by Braun and Clarke [35] is a flexible qualitative method not tied to any pre-existing theory, making it especially suitable for exploratory research. The process followed six phases: (1) familiarization with the data, (2) generation of initial codes, (3) identification of themes and subthemes, (4) revision of themes and subthemes, (5) definition and naming of themes and subthemes through consensus among researchers, and (6) final reporting.

Coding was conducted by two researchers independently. Any discrepancies in code assignment were resolved through discussion between both coders until full agreement was reached, and a third researcher reviewed the final codebook and the allocation of meaning units to codes. No qualitative analysis software was used. The analysis was organized using Microsoft Excel, whereby the codes were progressively classified into categories, subthemes, and main themes.

Field notes were analysed in parallel with the interview transcripts and were used to contextualise participants’ accounts, support the interpretation of meaning units, and contribute to the refinement and validation of the emerging themes, although the themes were primarily derived from the interview data.

### 2.5. Quality and Methodological Rigor

To ensure the study’s rigor, the four criteria proposed by Lincoln and Guba [36,37] (credibility, transferability, dependability, and confirmability) were applied. Various strategies were employed to strengthen the trustworthiness of the findings, including method and investigator triangulation during analysis (via double coding), participant validation of results, detailed description of the study context and process, and sustained researcher reflexivity throughout the investigation.

### 2.6. Research Team and Reflexivity

The research team comprised four occupational therapists, a nurse researcher, and a physiotherapist. The primary researcher (A.C.-M.) works as an occupational therapist at the nursing home where the study was conducted, providing direct contextual knowledge of the care environment. The remaining five researchers work as academics and have extensive experience in qualitative research. In addition, five of the six researchers have experience in geriatric care.

All in-depth interviews were conducted by the primary researcher. Given her dual role as care professional and interviewer within the same facility, several measures were implemented to minimise power imbalance and reduce the risk of socially desirable responses. This dual role also introduced reflexive considerations regarding its potential influence on recruitment processes, participants’ willingness to take part, interview dynamics, and the degree of disclosure during interviews, as well as on the interpretation of findings. Prior to data collection, participants were explicitly informed, both verbally and through the informed consent process, that their participation was entirely voluntary and would have no bearing on the care they received. It was also explicitly communicated that their responses would not affect their participation in daily activities, care plans, or any aspect of their life within the nursing home, in order to further reduce perceived coercion and social desirability bias. Interviews were conducted in a non-hierarchical manner: the primary researcher explained clearly at the outset that neither positive nor negative responses were sought, that the aim was to understand participants’ perspective as honestly as possible, and that they were encouraged to speak freely regardless of whether their experience had been positive or negative. Reflexivity was further maintained through regular research team debriefing meetings and a reflective journal kept by the primary researcher throughout the data collection and analysis process.

The research team acknowledged that the primary researcher’s dual role as occupational therapist involved in treating participants at the time of the study and interviewer could influence participant responses and data interpretation. Participants may have perceived her as a member of staff involved in their care, potentially increasing the likelihood of socially desirable responses. To minimise this risk, participants were repeatedly reminded that participation was voluntary, that responses would remain confidential, and that their care would not be affected by participation or by the content of their interviews. Reflexive discussions and field notes were used throughout data collection and analysis to critically examine how the primary researcher’s familiarity with the setting and prior assumptions might influence the interpretation of the findings.

In addition, the research team engaged in structured reflexive discussions throughout the analytic process to critically examine how the primary researcher’s positionality within the institution may have shaped data generation and interpretation.

These reflexive discussions also informed the analytic process. For example, during the initial stages of coding, some positive accounts regarding the nursing home’s management were interpreted cautiously because the primary researcher’s dual role raised the possibility that participants might have moderated their responses due to social desirability. However, reviewing the interview transcripts together with the field notes and discussing these narratives within the research team revealed that many of the same participants simultaneously acknowledged the necessity of the restrictive measures while openly criticised specific aspects of their implementation. For example, one participant stated, *“I came to terms with not having visitors. It had to be that way. At first, it was hard to accept, but I got used to it”* (Participant 8), whereas another explained, *“I was at war with [name of caregiver] because she kept telling me, ‘You can’t go out today’… I was really upset about being stuck inside”* (Participant 12). This led the team to refine the initial interpretation and understand these accounts as reflecting an ambivalent experience, in which residents simultaneously recognised the protective value of the measures while expressing frustration over the loss of autonomy.

A second example emerged during the analysis of participants’ narratives concerning access to healthcare. Initial coding grouped these accounts under barriers to healthcare access. However, reflexive discussions highlighted that several participants spontaneously framed these experiences as being denied healthcare specifically because of their age, despite this issue not being explicitly explored in the interview guide. Narratives such as *“It made me angry to hear that they were letting older people die to prioritize the young; we have the same right to be saved”* (Participant 2) and *“Just because of our age, we weren’t treated. That’s a disgrace”* (Participant 20) prompted the research team to re-examine these excerpts. Consequently, these narratives were re-coded, leading to the development of the distinct subtheme *“Not treated because of our age”*, which more accurately captured participants’ perceptions of age-based discrimination rather than barriers to healthcare access alone.

### 2.7. Compliance with Ethical Standards

The study was approved by the Ethics Committee of the Universidad Rey Juan Carlos (code 0105202011020) and was authorized by the nursing home where the study took place. Written informed consent was obtained from all participants.

## 3. Results

The study included 24 participants, whose clinical and sociodemographic data are presented in Table 2. The predominant profile consists of older widowed women with multiple comorbidities who have preserved cognitive function and have resided in the nursing home for an average of approximately 4 to 5.5 years.

Two main themes were identified: (1) Necessary to feel safe, but unpleasant: accepting the restrictive measures, which included two subthemes: Accepting the measures and Better safe, even if unpleasant; and (2) Barriers to healthcare: abandonment, fear, and age-based exclusion, which included three subthemes: Neglect and abandonment by healthcare system; The residence as a “bubble” and fear of hospital transfer; and Not treated because of our age.

### 3.1. Theme 1: Necessary to Feel Safe, but Unpleasant: Accepting the Restrictive Measures

#### 3.1.1. Accepting the Measures

Most participants indicated that the protective measures implemented in the nursing home, such as regular disinfection, temperature checks, and routine testing, were perceived as necessary and appropriate for safeguarding their health. Some participants noted that it took them a while to fully accept these restrictions.

*“I recognize it’s for our own good. I accept it, and I think it’s the right thing to do.”*

(Participant 2)

*“I came to terms with not having visitors. It had to be that way. At first, it was hard to accept, but I got used to it.”*

(Participant 8)

The restriction on family visits was among the most emotionally challenging aspects of the pandemic measures, generating feelings of sadness and longing even among those who ultimately accepted them as necessary.

*“It makes me very sad, because I am desperate to see my children.”*

(Participant 3)

*“I understand and accept that we cannot see our families, but if it is for everyone’s health and wellbeing, then so be it.”*

(Participant 15)

Although most participants ultimately accepted the restrictions, some expressed disagreement with specific rules, describing them as absurd or contradictory. These included not being allowed in the outdoor patio or being confined to the residence while the general population was permitted to go outside. Such inconsistencies led some residents to question or resist the measures imposed on care homes, particularly when they clashed with the measures imposed by the government on the general population.

*“Not being allowed to leave the premises. I didn’t understand it. It was the same patio. Those things were absurd.”*

(Participant 5)

*“There were measures I didn’t like. I had to stay locked up while people were out on the streets, even knowing what was going on. It wasn’t fair… The government issued some silly and contradictory rules, and I complained to you about it.”*

(Participant 11)

*“I was at war with* [name of caregiver] *because she kept telling me, ‘You can’t go out today,’ and I was really upset—that was the hardest part for me, having to accept those measures so suddenly… I really let* [name of caregiver] *have it because at first I was really upset about being stuck inside”*

(Participant 12)

These findings suggest that residents’ acceptance of restrictive measures was based primarily on trust in the facility and the perception that their health was being actively protected.

#### 3.1.2. Better Safe, Even if Unpleasant

Most participants generally perceived the internal protocols, such as temperature screening, disinfection routines, and mandatory mask use, as effective in preventing infection. These measures contributed to a sense of safety and control.

*“Protocols were followed to the letter. The care staff did everything right: they checked temperatures when entering and leaving, disinfected their shoes, and kept their hands clean. Everything was handled properly.”*

(Participant 5)

*“I saw the whole cleaning and control process. I felt like the virus wasn’t going to get in because they were doing everything so well.”*

(Participant 17)

Most participants also described how the efforts taken by the staff, such as daily monitoring, visitor restrictions, and strict hygiene protocols, offered them reassurance and peace of mind. Some even noted feeling safer inside the facility than outside in the general community.

*“Seeing them take so many precautions every day made me feel calm and safe.”*

(Participant 15)

*“It reassured me to see that more measures were being taken here than outside.”*

(Participant 22)

Still, some participants acknowledged that the strictness of the rules made them burdensome—for both residents and staff. Despite the discomfort, they understood the measures were implemented for their well-being.

*“The measures were welcome, even if unpleasant. I didn’t like them, but they were for our own good.”*

(Participant 1)

*“The restrictions were hard for everyone—for us and for you.”*

(Participant 2)

Although participants expressed mixed emotions, many expressed unease when restrictions were eased after three months of strict confinement. Most of them believed that lifting the measures increased the risk of infection.

*“When people started coming back in, I didn’t like it. The virus could come in again and we’d all get sick. That was a huge source of anxiety.”*

(Participant 18)

*“We should go back to the way things were, being more careful and keeping the same rules. I think we’d be better and more at ease.”*

(Participant 21)

In summary, the participants’ narratives indicate that, although the restrictive measures generated discomfort and tension, the residents ultimately perceived them as effective and necessary.

### 3.2. Theme 2: Barriers to Healthcare: Abandonment, Fear, and Age-Based Exclusion

#### 3.2.1. Neglect and Abandonment by Healthcare System

Most participants described that, during this period, they were unable to access medical resources such as primary care or specialized consultations (e.g., oncology or psychiatry) under normal conditions. This lack of medical attention led to feelings of neglect, helplessness, abandonment, sadness, and even anxiety and anger, as they perceived that they were not receiving adequate care due to the system being overwhelmed.

*“It made me feel unwell and angry; not only COVID patients need care, there are many of us who are ill and we were being ignored”*.

(Participant 4)

*“I feel abandoned for not being able to see the doctor whenever I need to”*.

(Participant 5)

They also noted that during this time, healthcare focused primarily on mitigating the effects of COVID-19, leaving behind follow-ups and treatments for other conditions such as cancer, Parkinson’s disease, or psychiatric and neurological issues. Most participants expressed concern that while COVID-19 might not kill them, the lack of care for other diseases could.

*“It affects me not being able to go, because I’m recovering from cancer and I felt that I wouldn’t die from COVID, but I might if I didn’t have follow-up care”*.

(Participant 5)

*“It’s true that COVID came first… but surgeries like mine couldn’t be performed”*.

(Participant 7)

Conversely, some participants accepted the inability to attend medical appointments, understanding the context, and some continued to follow the medical advice they had received previously while waiting for the situation to improve.

*“I saw there was no other option, so I accepted it, and I’ll go when I can”*.

(Participant 7)

*“It didn’t affect me not seeing him [the doctor], because I’m still following his advice”*.

(Participant 16)

These experiences reveal that the suspension of routine and specialized medical care during the pandemic generated a profound sense of abandonment among residents, representing a critical view of their experiences during the crisis, beyond the restrictive measures themselves.

#### 3.2.2. The Residence as a “Bubble” and Fear of Hospital Transfer

Some participants expressed that they did not want to attend medical consultations because they considered themselves high-risk individuals and feared that becoming infected would seriously endanger their health. They preferred to remain in the residence, which they perceived as a safe, closed environment. A few participants even said they would rather die peacefully in the residence than leave it to attend a consultation.

*“I’m aware that going outside the residence increases my risk of infection”*.

(Participant 16)

*“There’s less risk of illness here, it’s like a closed circle where we’re safer, that’s why I didn’t want to leave”*.

(Participant 20)

Another aspect highlighted was the fear associated with possible hospital transfers. Several participants said they prayed not to fall ill and require hospitalization, as they felt hospitals were unable or unwilling to make the same effort to save older adults. They believed they would not survive once admitted.

*“Every morning I prayed not to get sick so you wouldn’t have to send me to the hospital… I knew I wouldn’t come back, because they saved the young ones first, and that’s terrifying”*.

(Participant 1)

*“I felt like a cow going to the slaughterhouse, that’s how I felt going to the hospital”*.

(Participant 19)

These narratives illustrate how fear of infection transformed the residence into a safe space, shaping residents’ attitudes towards medical care outside the facility and highlighting the psychological cost of institutional confinement.

#### 3.2.3. Not Treated Because of Our Age

Finally, participants spoke of the differential treatment received from healthcare services based on age. Ageism emerged as an inductive theme in the context of discussions on access to healthcare services. Although it was not directly prompted by the interview guide, several participants spontaneously described experiences and perceptions consistent with age-related discrimination in healthcare access. On the one hand, they acknowledged it was reasonable to prioritize younger individuals, but being left to die evoked sadness, anger, and a sense of injustice. Many were surprised by this inequality, especially after having spent their lives fighting for rights they now felt were being denied. They could not understand how age alone could justify being left without care during a time of system overload.

*“It made me angry to hear that they were letting older people die to prioritize the young; we have the same right to be saved”*.

(Participant 2)

*“Just because of our age, we weren’t treated. That’s a disgrace”*.

(Participant 20)

These experiences point to a perceived system of age-based exclusion from healthcare during the pandemic, an experience that residents interpreted not merely as a practical consequence of resource scarcity, but as a fundamental violation of their rights.

## 4. Discussion

The findings suggest that most participants accepted the measures implemented by the nursing home, considering them necessary and aimed at their protection, which also contributed to feelings of calm and well-being. This result aligns with that reported by Sims et al. [15], who found that many residents expressed a sense of gratitude linked to safety and well-being despite restrictive measures implemented in care homes. Nevertheless, other studies [38] highlight more negative experiences, indicating that such restrictions were associated with loneliness, sadness, depressive symptoms, and feelings of helplessness among residents. These findings suggest that, while acceptance was common, adaptation to the measures was not uniform and often entailed significant emotional distress and ambivalence. This apparent contradiction suggests that residents were not simply complying with institutional rules, but actively negotiating the tension between personal autonomy and collective safety. Acceptance of the measures may therefore reflect a process of adaptation in which protection from infection was perceived as outweighing the loss of freedom and social contact. The need to balance physical protection and psychological well-being in long-term care settings has been highlighted in the literature, which calls for future emergency protocols to consider not only infection control but also the preservation of residents’ mental health and social connectedness [39,40].

However, this acceptance was not free from tension. The restriction of family visits was one of the most difficult measures for participants to cope with due to its emotional impact. Previous studies have shown that distancing and visiting restrictions generated anxiety, grief, and severe stress among residents and their families [39]. During the COVID-19 pandemic, visitation restrictions were associated with increased loneliness, depression, and mood disturbances, particularly among residents without cognitive impairment, similar to those included in our study. However, some studies reported no significant adverse effects, or even reductions in depressive symptoms, possibly due to strategies implemented to maintain social engagement [38]. Moreover, the perception that some internal restrictions were stricter than those applied in the general community generated a sense of perceived injustice among the participants in our study. These perceptions may reflect residents’ awareness that, despite living in a care environment, they remained individuals with social and relational needs. Restrictions were therefore experienced not only as infection-control measures but also as limitations on meaningful relationships and personal autonomy. This finding is consistent with previous research indicating that residents were effectively more isolated from the outside world, as care home restrictions limited contact and mobility to a greater extent than in the general community [40].

Another critical issue was the limited access to medical services. Participants expressed feelings of helplessness and abandonment, in line with Su [41], who, based on Maslow’s hierarchy of needs [42], argues that the absence of access to basic medical care can compromise psychological well-being, particularly in vulnerable populations such as older adults. Reduced contact with healthcare professionals may have further contributed to these experiences, as previous research has associated limited interaction and access to care with increased anxiety, stress, and psychological distress among older adults [43]. Beyond the practical consequences of delayed care, participants appeared to interpret reduced healthcare access as a symbolic form of abandonment. The inability to obtain medical attention challenged their sense of security and reinforced perceptions of vulnerability during the pandemic.

Fear of infection also emerged as a significant barrier to seeking medical care. Many participants perceived leaving the residence as a health risk, which is consistent with findings by Pujolar [44] and Czeisler [45], who documented a reduction in healthcare demand due to fear of contagion. This pattern of avoidance of seeking medical care or accessing healthcare services was also identified among older adults, where higher perceived risk and pandemic-related concerns were associated with delayed or avoided healthcare utilization [46].

The concept of the “bubble” frequently appeared in the participants’ narratives, referring to the residence as a safe and isolated space. This perception aligns with Lood [47], who described nursing home residents as experiencing a form of disconnection from the outside world that was both protective and restrictive. While residents felt cared for and shielded from the virus, they also reported a loss of freedom and social contact. Other studies also interpret residential facilities as protective environments, albeit at the cost of social isolation [40].

Fear of hospitalization was another relevant finding, linked to uncertainty, stigmatization, and the perception of hospital overcrowding. Participants’ narratives are consistent with studies that describe fear of the unknown and judgment from others as key stressors during the pandemic [48,49], as well as concerns related to triage decisions and equitable access to care, including the potential denial of healthcare in contexts of resource scarcity [50].

Finally, the perception of inequity in access to healthcare services based on age elicited ambivalent reactions. While some participants acknowledged the logic of prioritizing younger individuals, consistent with Reeskens [51], who showed that age is often considered a legitimate criterion for allocating scarce healthcare resources, others expressed frustration and sadness at feeling excluded. This ambivalence reflects the tension identified in previous research between the normative acceptance of prioritization principles and concerns about fairness. This experience was interpreted as a form of age-based discrimination, or ageism, a phenomenon widely documented in the literature and exacerbated in contexts of critical resource scarcity such as ICU beds and ventilators [52,53].

These findings should be contextualized within the broader literature on ageism in healthcare and long-term care ethics. Age-based discrimination is a phenomenon that predates the COVID-19 pandemic, manifesting as reduced access to treatments, diagnostic procedures, and specialist care for older adults across healthcare systems [52,53,54]. The pandemic did not generate these inequities but intensified their visibility and impact, particularly in institutional settings with highly vulnerable populations [18]. From a rights-based perspective, such experiences reflect not only resource constraints but also structural shortcomings in safeguarding residents’ rights to dignity, autonomy, and equitable care [21]. International frameworks emphasize that long-term care should adhere to person-centred, rights-based principles, ensuring that emergency responses do not disproportionately compromise the entitlements of older adults, irrespective of crisis severity [21].

These findings have direct implications for the management of nursing homes and healthcare policy. Future emergency protocols must ensure continuity of care, including access to specialist consultations, and incorporate structured mechanisms for psychological support for residents during periods of heightened restriction. At the policy level, triage criteria must ensure that age alone does not determine exclusion from care, and rights-based emergency frameworks must be established that explicitly protect residents’ rights.

The findings should also be interpreted within the broader context of community and institutional support during public health emergencies. Although participants primarily focused on their experiences within the nursing home and the healthcare system, the COVID-19 pandemic highlighted the critical role of coordination between long-term care facilities, local health authorities, primary care services, and social care systems. Previous studies have shown that healthcare system organization, resource allocation, and institutional support were key factors influencing the quality of care provided to older adults during the pandemic [3,21]. Furthermore, evidence suggests that collaborative approaches involving healthcare providers, public authorities, and residential care facilities may help reduce the vulnerability of nursing home residents and support the implementation of more equitable and person-centred care strategies during crises [21,40].

The experiences reported by participants also underscore the importance of strengthening preparedness plans for future health emergencies. Future contingency strategies should ensure continuity of medical care, maintain social and family connections whenever possible, provide psychological support for residents, and establish clear coordination mechanisms between nursing homes and healthcare services. Previous research has emphasized that emergency responses in long-term care setting should balance infection-control measures with residents psychological well-being, dignity, and fundamental rights [21,40]. Strengthening these preparedness frameworks may improve the resilience of long-term care facilities and enhance their capacity to respond effectively to future public health crises.

This study has several limitations. First, its qualitative design limits the generalizability of the findings to other populations or care settings. Second, data collection was carried out in a single nursing home, which restricts the diversity of experiences and may reflect institution-specific characteristics. Third, data collection was conducted over an extended period (May 2020 to December 2021), during which the pandemic context evolved considerably, including changes in restrictive measures, healthcare access, mortality rates, and the implementation of vaccination programmes. These changes may have influenced participants’ recollections and perceptions of their experiences. However, no substantial differences were observed in participants’ narratives according to the timing of the interviews, as similar perceptions regarding restrictive measures, access to healthcare services, and emotional impact emerged throughout the study period. Finally, it was not intended to represent the full spectrum of nursing home residents, but rather to ensure that participants had fully preserved cognitive function and were able to provide detailed, reliable, and reflective accounts of their experiences during the interviews. An MMSE score of ≥27 was therefore selected to minimize the potential influence of even mild cognitive impairment on participants’ recall, comprehension, and narrative ability. This criterion may have excluded some residents with normal cognitive functioning according to other MMSE cutoffs and may therefore limit the transferability of the findings.

In order to mitigate these limitations, the research process has been described in detail, along with the characteristics of the research team and the context in which the study was conducted, with the aim of facilitating the transferability of the results to other contexts with similar characteristics, as outlined in the quality criteria established by Lincoln and Guba [36].

In summary, participants’ experiences during COVID-19 reflect a balance between accepting protective measures and facing emotional challenges, including limited family contact, restricted medical access, and perceived age-based inequities. These findings highlight the need to include residents’ perspectives in emergency planning, ensuring both health protection and psychological well-being in long-term care settings.

## 5. Conclusions

These findings show that older adults in residential care experienced the COVID-19 pandemic as a balance between feeling protected by restrictive measures and experiencing significant emotional distress associated with social isolation, reduced family contact, limited participation in daily activities, and difficulties accessing healthcare services. Participants also reported feelings of fear, vulnerability, abandonment, and age-based inequity during this period. While the protective measures were generally accepted, they also caused emotional distress due to isolation, reduced family contact, and limited medical access. The interplay of fear, abandonment, and perceived age-based inequity highlights the need to reassess institutional strategies from an ethical, rights-based approach. Health responses must ensure not only physical safety but also psychological well-being and fairness for older adults in emergency contexts.

These findings suggest that incorporating residents’ perspectives is essential to balance protection, emotional support, and equitable care in long-term care settings. This study provides key evidence to identify strengths and weaknesses in emergency management within long-term care settings, while also documenting how users were affected through literal narratives of the feelings and experiences that emerged during this period. This approach offers valuable insights for designing more effective protocols, optimizing staff training, and ensuring the availability of critical resources, thereby enhancing the resilience of these institutions in the face of future health crises or other emergencies.

## Figures and Tables

**Table 1:** Semi-structured interview guide.

Research Area	Questions
Opening Question	What has your experience been like living in the nursing home during the healthcare crisis resulting from COVID-19?
Nursing Home Management of the Pandemic	How do you assess the management of the pandemic by the nursing home? How do you assess the measures taken by the nursing home? How do you assess the performance of the staff? What impact has the management of the nursing home, the measures taken and the performance of the staff had on your experience during the pandemic?
Access to medical/health services internal/external to the nursing home	Has the healthcare you receive inside or outside the nursing home been affected in any way? How? If you have been affected, how has the inability to access medical resources impacted you? How did you feel about the possibility of having to attend a health consultation/appointment outside the nursing home? How do you evaluate the solutions that have been taken (e.g., telephone consultations) in view of the impossibility of going to medical centers?
Aspects related to illness and death	Have you had people close to you who get sick or passed away because of COVID-19? If so, how has it made you feel? At any time have you thought about the possibility of getting sick or dying from COVID-19? If so, how has it made you feel?

**Table 2:** Clinical and sociodemographic characteristics of participants.

Sex (female/male)	18/6
Age (mean ± SD)	82.1 ± 8.9
Marital status	Widowed: 13
	Single: 6
	Divorced: 2
	Married: 3
MMSE score (mean ± SD)	28.7 ± 0.8
Length of stay (months, mean ± SD)	57.5 ± 9.4
Types of illnesses	Metabolic diseases: 8
	Cardiovascular diseases: 6
	Musculoskeletal/locomotor diseases: 5
	Neurological diseases: 4
	Respiratory diseases: 4
	Sensory/visual/auditory disorders: 4
	Mental disorders: 2
	Cancer/neoplasms: 2
	Infectious diseases: 2
	Digestive/renal diseases: 2
	Addictive behaviors: 2
	Blood/hematologic disorders: 1

MMSE: Mini-Mental State Examination; SD: standard deviation.

## Data Availability

The data presented in this study are available on request from the corresponding author due to ethics and legal restrictions.
